# Morphological and Compositional Studies on Al/Ti/TiN/Si, Al/TiN/Si, Al/W/Si, Al/WN/Si Systems to Test the Diffusion Barrier Properties of Nanoscale-Thick Layers between Al and Si

**DOI:** 10.3390/mi12080849

**Published:** 2021-07-21

**Authors:** Maria Censabella, Cristina Drago, Brunella Cafra, Paolo Badalà, Anna Bassi, Giovanni Piccitto, Salvatore Mirabella, Maria Grazia Grimaldi, Francesco Ruffino

**Affiliations:** 1 Dipartimento di Fisica e Astronomia “Ettore Majorana”, Università di Catania, via S. Sofia 64, 95123 Catania, Italy; maria.censabella@ct.infn.it (M.C.); cri-rocker@hotmail.it (C.D.); giovanni.piccitto@ct.infn.it (G.P.); salvatore.mirabella@ct.infn.it (S.M.); mariagrazia.grimaldi@ct.infn.it (M.G.G.); 2 CNR-IMM, via S. Sofia 64, 95123 Catania, Italy; 3 STMicroelectronics, Zona Industriale Stradale Primosole 50, 95121 Catania, Italy; brunella.cafra@st.com (B.C.); paolo.badala@st.com (P.B.); anna.bassi@st.com (A.B.)

**Keywords:** Al, Si, diffusion barrier, W, WN, TiN

## Abstract

In this work, an investigation of the properties of nanoscale-thick Ti/TiN, TiN, W, WN layers as diffusion barriers between Si and Al is carried out in view of Si-based electronic applications. Heat treatments were performed on the samples to activate interdiffusion between Si and Al. Changing annealing time and temperature, each sample was morphologically characterized by scanning electron microscopy and atomic force microscopy and compositionally characterized by Rutherford backscattering analysis. The aim is to evaluate the efficiency of the layers as diffusion barriers between Si and Al and, at the same time, to evaluate the surface morphological changes upon annealing processes.

## 1. Introduction

The demand for miniaturization in integrated circuits (ICs) has grown exponentially over the years, as predicted by Moore’s law [1,2,3]. Due to its multiple properties, silicon (Si) is the pivotal material around which the microfabrication processes of integrated circuits evolve. The search for metals capable of contacting the Si in ICs is a fundamental field of research for technological advances. The metal by far mainly used for Si metallization is aluminum (Al) [1,2,3,4], which, due to its many properties (such as low resistivity, excellent adhesion to Si and SiO_2_), ensures good ohmic contact. There is, however, a serious problem in the Al/Si contact: at typical temperatures used in the microfabrication processes of metal–oxide–semiconductor field-effect transistors (MOSFETs) and integrated circuits (450–500 °C), the solubility of Si in Al is 0.5% and the Al/Si system becomes unstable, giving rise to a substantial interdiffusion between Al and Si [4,5,6,7]. Si diffuses in Al until the solubility limit is reached and, for example, the diffusion length, after a heat treatment at a temperature of 450 ° C, for a time of 30 min, is about 40 μm [1]. The interdiffusion between Al and Si causes the formation of cavities in Si, which are, then, filled by Al. This leads to the formation of Al spikes, which can cause short circuits by penetrating into the underlying Si layer. To avoid this phenomenon, a sharp separation between Al and Si is required; thus, material layers acting as diffusion barriers between Si and Al are strongly required [1,4,5,6,7]. Hence, the study of the properties of materials acting as diffusion barriers between Al and Si has been extensively covered by the literature in the past years. An ideal diffusion barrier should be characterized by some fundamental properties, such as high electrical conductivity, chemical and mechanical stability till the device reaches processing temperatures, good adhesion, and feasibility of fabrication. The typical materials fulfilling these requirements are refractory metals, having a high melting point (so as to minimize the formation of grain boundaries during the deposition step) and high chemical stability. Furthermore, impurities are often added to these metals, such as O, N, and C, which on the one hand increase the barrier stability and, on another hand, saturate the grain boundaries which are the main diffusion paths between Al and Si [1,4,5,8,9,10,11,12,13,14,15,16,17,18,19,20]. These materials act as “sacrificial barriers”: the refractory metal reacts with the above-placed Al film ensuring that the formation of the corresponding compound avoids the diffusion of Al into the barrier and, subsequently, into the underlying Si (usually these material barriers do not react with Si because such reactions would require much higher temperatures than those used for the device processing). An example of such a barrier is a Ti layer between Al and Si [5]: at 450 °C, Ti does not react with Si; however, it reacts with Al, forming a continuous and uniform layer of TiAl_3_ which inhibits the Al diffusion towards the Si (the substrate). As long as there is even a very thin layer of Ti that is not consumed by the formation of TiAl_3_, the contact maintains its structural integrity. When the Ti layer is completely consumed, the diffusion barrier effect of the layer ends and further heat treatments will lead to a degradation of the contact. The use of Ti layers as diffusion barriers has been widely discussed in the literature. However, in order to improve its performance, the addition of N as an impurity to the simple Ti layer, forming a TiN compound, was found to be an effective strategy. However, the efficiency of TiN as a diffusion barrier was found to be strongly dependent on stoichiometry and on the deposition methodology, a phenomenon even more evident when using thin films, since the latter are normally synthesized in non-equilibrium conditions. W and WN constitute other materials exploited as diffusion barrier layers between Al and Si [5,7,18,19,20]; as previously mentioned, the addition of impurities such as N increases the chemical stability of W and saturates the grain boundaries, inhibiting Al grain boundaries diffusion. WN also belongs to the family of refractory metals and the structure of WN films strongly depends on the deposition parameters: for example, the structure of sputter-deposited WN films can be changed from the crystalline phase to the amorphous phase by the deposition power with a major impact on the layer performance as a diffusion barrier between Al and Si [18,19,20].

In this regard, the continuous scaling of devices also leads to the continuous reduction of the thickness of the diffusion barrier layers; it is of paramount importance to study the temperature-dependent properties of materials as diffusion barriers between Al and Si when reduced to very thin films so as to find the best barrier materials and best operation conditions.

Starting from these considerations, in this work, we report on experimental studies on the properties of four different materials to be used as nanoscale-thick diffusion barriers between Al and Si. In particular, four different types of samples are examined; therefore, four different diffusion barriers between Al and Si are observed: Ti-TiN, TiN, W and WN. The aim of the work is to evaluate their efficiency and to establish, through heat treatments and corresponding morphological and elemental characterizations, which of these materials can most effectively counteract the problem of interdiffusion between Al and Si.

## 2. Experimental

The samples studied in this work, as well as the different diffusion barriers, are four, respectively:

Sample 1: Al(100 nm)/Ti(10 nm)/TiN(40 nm)/Si
Sample 2: Al(100 nm)/TiN(40 nm)/Si
Sample 3: Al(100 nm)/W(40 nm)/Si
Sample 4: Al(100 nm)/WN(40 nm)/Si

**Figure 1:**
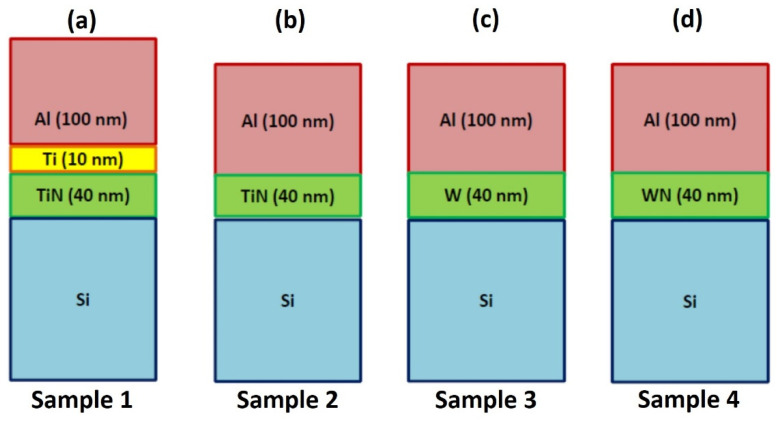
Picture reporting the scheme of the four samples analyzed in this work. (**a**), Sample 1: Al(100 nm)/Ti(10 nm)/TiN(40 nm)/Si, (**b**), sample 2: Al(100 nm)/TiN(40 nm)/Si, (**c**), sample 3: Al(100 nm)/W(40 nm)/Si, (**d**), sample 4: Al(100 nm)/WN(40 nm)/Si. In each sample, the Si is the substrate, the surface Al layer is the electrical contact and the layers between Al and Si are the materials used as diffusion barriers.

Their structure is, schematically, reported in Figure 1.

In particular, regarding sample 1 and sample 2 the diffusion barriers (i.e., the Ti/TiN layers in sample 1 and the TiN layer in sample 2) were deposited on the Si substrate by the sputtering deposition technique, while regarding sample 3 and sample 4 the diffusion barriers (i.e., the W layer in sample 3 and the WN layer in sample 4) were deposited on the Si substrate by thermal evaporation. Finally, the 100 nm-thick Al surface layer was deposited on all the barrier layers by the sputtering deposition technique. Then, we proceeded to heat treatments on the samples, increasing temperature and time to mimic the thermal processes to which these structures are typically subjected during the production steps of real commercial devices, after depositions. Table 1 summarizes all the combinations of annealing temperature and time for the samples and the corresponding labels which were associated to the samples after the annealing processes.

The thermal treatments were performed by using a Carbolite Horizontal Furnace under dry N2 flux (1 lpm). The surface morphology of the Al layer in each sample, before and after the annealing processes, was analyzed by scanning electron microscopy (SEM, Carl Zeiss Microscopy, New York, NY, USA) and atomic force microscopy (AFM, Billerica, MA, USA). SEM analyses were performed by using a Zeiss FEG-SEM Supra 25 Microscope operating at 5 kV. The SEM images were analyzed by the Gatan Digital Micrograph software. AFM analysis was performed by using a Bruker-Innova microscope operating in high-amplitude mode. Concerning this analysis, ultra-sharpened Si tips were used (MSNL-10 from Bruker Instruments, Billerica, MA, United States, with anisotropic geometry, radius of curvature ~2 nm, tip height ~2.5 μm, front angle ~15°, back angle ~25°, side angle ~22.5°, nominal spring constant of 0.07 N/m). The Si tips were substituted as soon as a resolution loose was observed during the AFM image acquisition. The AFM images were analyzed by using the SPMLABANALYSES V7.00 software to extract, in particular, values for the root mean square (RMS) of the Al layer surface. Compositional analysis of the samples was performed by Rutherford backscattering spectrometry (RBS, High Voltage Engineering Europa BV, The Netherlands) measurements, in particular to investigate possible interdiffusion between Al and Si through the diffusion barriers after the annealing processes. The RBS analysis was performed by irradiating the samples with a beam (about 1 mm in diameter) of ^4^He^+^ ions having energy of 2 MeV and revealing the backscattered ions at 165°. However, in order to maximize the yield and amplify the signal of the various elements, the measurements were made by tilting the samples by an angle θ = 60° with respect to the direction of the incident beam. SIMNRA simulations [21] concerning the RBS analysis suggested that the 40 nm thickness for the TiN, W, and WN layers between Al and Si is the smaller thickness at which the RBS spectra, in our best experimental conditions, can provide depth-dependent compositional differences upon annealing. In these experimental conditions, possible depth-dependent compositional differences, upon annealing, would be not detectable by the RBS spectra, according to the SIMNRA results.

## 3. Results and Discussions

### 3.1. Surface Morphological Analysis

**Figure 2:**
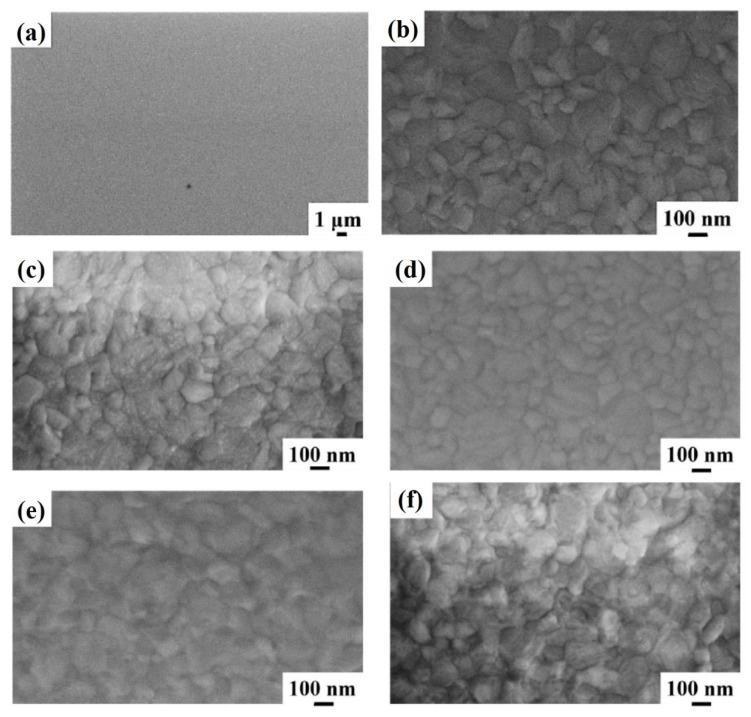
Representative plan-view SEM images of the Al surface for the sample: (**a**,**b**) 1A (Si/TiN/Ti/Al) with increasing magnification from (**a**) to (**b**); (**c**) 1E (Si/TiN/Ti/Al + 350 °C 30 min); (**d**) 1F (Si/TiN/Ti/Al + 400 °C 15 min); (**e**) 1G (Si/TiN/Ti/Al + 450 °C 15 min); (**f**) 1H (Si/TiN/Ti/Al + 500 °C 15 min).

Figure 2 reports representative plan-view SEM images of the Al surface for the sample: (a)–(b) 1A (Si/TiN/Ti/Al) with increasing magnification from (a) to (b); (c) 1E (Si/TiN/Ti/Al + 350 °C 30 min); (d) 1F (Si/TiN/Ti/Al + 400 °C 15 min); (e) 1G (Si/TiN/Ti/Al + 450 °C 15 min); (f) 1H (Si/TiN/Ti/Al + 500 °C 15 min). Considering the image in Figure 2a, corresponding to the lowest magnification for sample 1A, a large-scale morphologically uniform Al surface can be observed. At higher magnifications (see Figure 2b), the granular structure of the sample surface is visible, indicating columnar grain growth for the Al layer as typical, as indicated by the classical “zone model” for growing films. The planar size of the grains is in the 100–300 nm range. Performing the annealing processes on sample 1A, by increasing the annealing temperature or annealing time, no morphological changes are evidenced by the SEM analysis for the Al surface, as shown by the high-magnification images schematically reported in Figure 2c–f.

**Figure 3:**
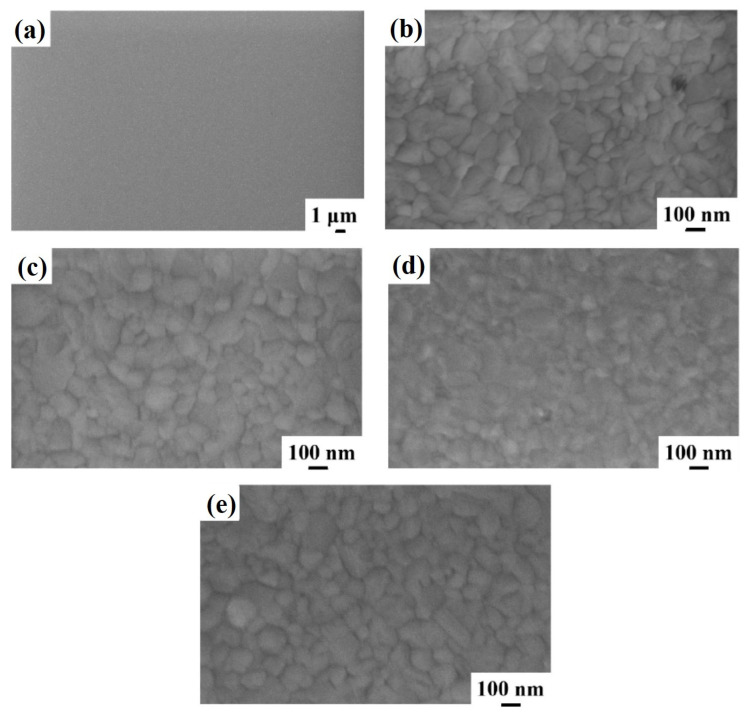
Representative plan-view SEM images of the Al surface for the sample: (**a**,**b**) 2A (Si/TiN/Al) with increasing magnification from (**a**) to (**b**); (**c**) 2E (Si/TiN/Al + 350 °C 30 min); (**d**) 2F (Si/TiN/Al + 400 °C 15 min); (**e**) 2G (Si/TiN/Al + 450 °C 15 min).

**Figure 4:**
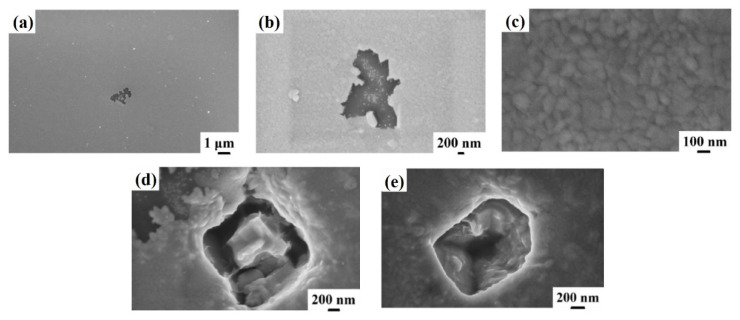
Representative plan-view SEM images of the Al surface for the sample: (**a**,**b**) 2H (Si/TiN/Al + 500 °C 15 min) with increasing magnification from (**a**) to (**c**). Regarding the same sample, (**d**) and (**e**) highlight the peculiarity of some surface defects appearing as holes in the Al layer.

Similar results are obtained for the surface morphology of the Al layer in the sample 2 till the annealing temperature of 450 °C. Figure 3 shows representative plan-view SEM images of the Al surface for the sample: (a)–(b) 2A (Si/TiN/Al) with increasing magnification from (a) to (b); (c) 2E (Si/TiN/Al + 350 °C 30 min); (d) 2F (Si/TiN/Al + 400 °C 15 min); (e) 2G (Si/TiN/Al + 450 °C 15 min). As in sample 1, the granular structure of the Al layer is visible (indicating a columnar grain growth). Performing the annealing processes on sample 2A, by increasing the annealing temperature or annealing time, no morphological changes are evidenced by the SEM analysis for the Al surface till 450 °C. A specific change occurs, instead, after annealing at 500 °C—see Figure 4, showing representative plan-view SEM images of the Al surface for sample 2H (Si/TiN/Al + 500 °C 15 min) with increasing magnification from (a) to (c). Regarding the same sample, (d) and (e) highlight the peculiarity of some surface defects appearing as holes in the Al layer. Considering the SEM images at low magnification (Figure 4a,b), we can see how the Al film undergoes considerable degradation, compatible with the fact that a certain amount of Al migrates through the underlying TiN barrier. This degradation is evidenced by some darker regions corresponding to retreating Al film or holes (see Figure 4d,e). These regions are, however, spatially localized: other regions of the Al layer are unaltered (see Figure 4c), showing the characteristic granular morphology of the untreated Al layer.

**Figure 5:**
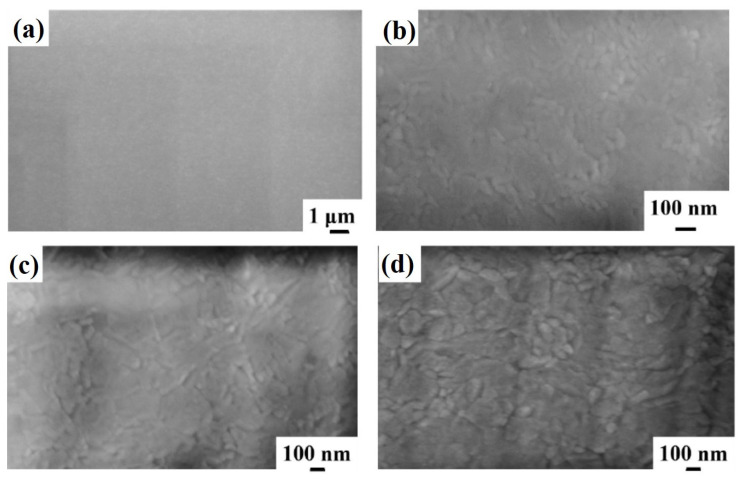
Representative plan-view SEM images of the Al surface for the sample: (**a**,**b**) 3A (Si/W/Al) with increasing magnification from (**a**) to (**b**); (**c**) 3E (Si/W/Al + 350 °C 30 min); (**d**) 3F (Si/W/Al + 400 °C 15 min).

Figure 5 reports representative plan-view SEM images of the Al surface for the sample: (a)–(b) 3A (Si/W/Al) with increasing magnification from (a) to (b); (c) 3E (Si/W/Al + 350 °C 30 min); (d) 3F (Si/W/Al + 400 °C 15 min). These analyses allow us to conclude that, till the 400 °C annealing temperature, no specific irregularities or surface defects occur in the Al layer, and the higher magnification images highlight the standard granular surface morphology of the layer.

**Figure 6:**
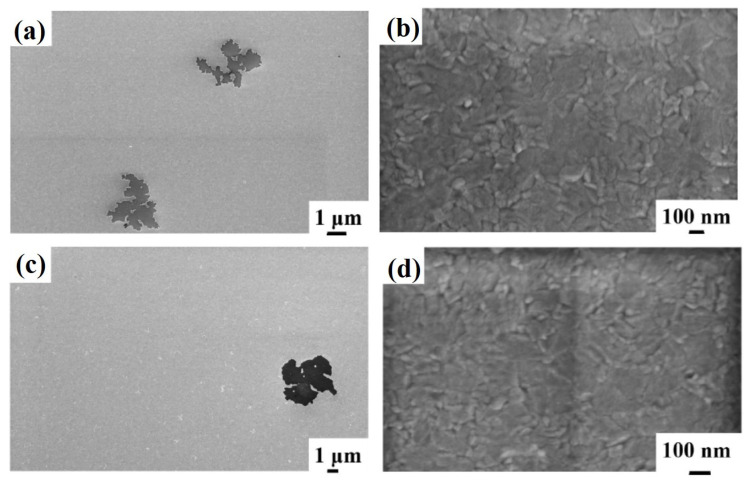
Representative plan-view SEM images of the Al surface for the sample: (**a**,**b**) 3G (Si/W/Al + 450 °C 15 min) with increasing magnification from (**a**) to (**b**); (**c**,**d**) 3H (Si/W/Al + 500 °C 15 min) with increasing magnification from (**c**) to (**d**).

Regarding sample 3, the surface morphology of the Al layer undergoes a significant change at the annealing temperatures of 450 °C and 500 °C. Figure 6 reports representative plan-view SEM images of the Al surface for the sample: (a)–(b) 3G (Si/W/Al + 450 °C 15 min) with increasing magnification from (a) to (b); and (c)–(d) 3H (Si/W/Al + 500 °C 15 min) with increasing magnification from (c) to (d). It is evident from the SEM images in Figure 6 that starting from the temperature of 450 °C, inhomogeneities and surface defects are found, presumably ascribed to the breaking of the Al film, indicating a possible migration of Al towards the substrate through the underlaying W layer. However, these surface inhomogeneities are spatially localized since other regions of the Al layer are unaltered (see Figure 6b–d), showing the characteristic granular morphology of the untreated Al layer.

**Figure 7:**
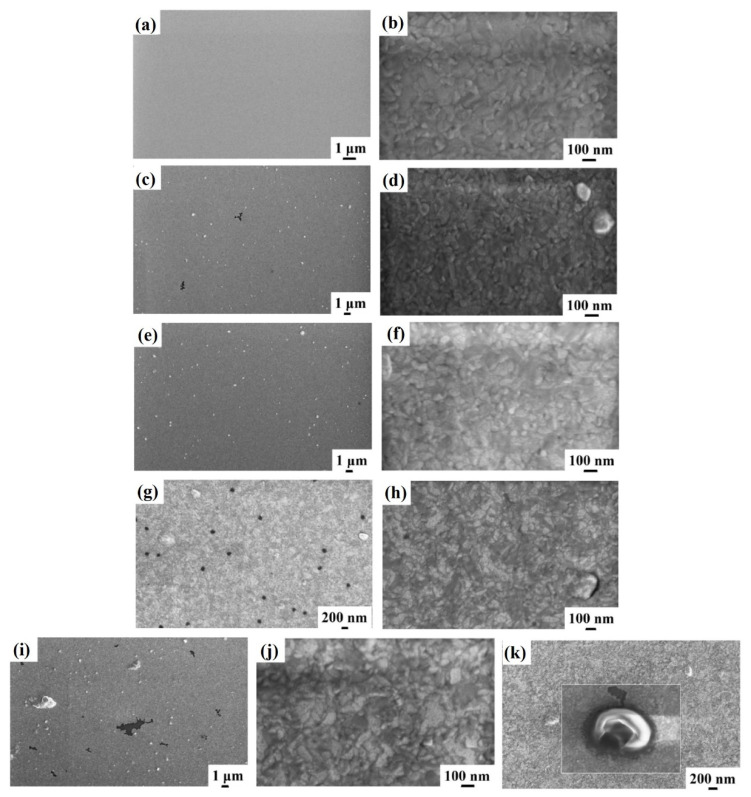
Representative plan-view SEM images of the Al surface for the sample: (**a**,**b**) 4A (Si/WN/Al) with increasing magnification from (**a**) to (**b**); (**c**,**d**) 4E (Si/WN/Al + 350 °C 30 min) with increasing magnification from (**c**) to (**d**); (**e**,**f**) 4F (Si/WN/Al + 400 °C 15 min) with increasing magnification from (**e**) to (**f**); (**g**,**h**) 4G (Si/WN/Al + 450 °C 15 min) with increasing magnification from (**g**) to (**h**); (**i**–**k**) 4H (Si/WN/Al + 500 °C 15 min) with increasing magnification and highlighting some surface features as a holes in the Al layer as in (**k**).

Finally, Figure 7 shows representative plan-view SEM images of the Al surface for the sample: (a)–(b) 4A (Si/WN/Al) with increasing magnification from (a) to (b); (c)–(d) 4E (Si/WN/Al + 350 °C 30 min) with increasing magnification from (c) to (d); (e)–(f) 4F (Si/WN/Al + 400 °C 15 min) with increasing magnification from (e) to (f); (g)–(h) 4G (Si/WN/Al + 450 °C 15 min) with increasing magnification from (g) to (h); (i)–(k) 4H (Si/WN/Al + 500 °C 15 min) with increasing magnification and highlighting some surface features as a holes in the Al layer as in (k). In this case, the typical granular surface morphology is, again, found and no morphological inhomogeneities are detectable till the annealing temperature of 300 °C. However, starting from the annealing temperature of 350 °C the surface of the Al shows evident defects and inhomogeneities’ formation, whose number increases by increasing the annealing temperature (see Figure 7 from (c) to (i)).

In particular, Figure 7k shows a high magnification image of a single surface defect indicating that it is compatible with the formation of a hole in the surface layer. However, these surface inhomogeneities are spatially localized since other regions of the Al layer are unaltered (see Figure 7b,d,f,h,j)), showing the characteristic granular morphology of the untreated Al layer.

The quantitative analysis of the change of the surface morphology of the samples upon annealing was performed by AFM analysis, so as to quantify the surface roughness of the Al layer by the RMS parameter.

**Figure 8:**
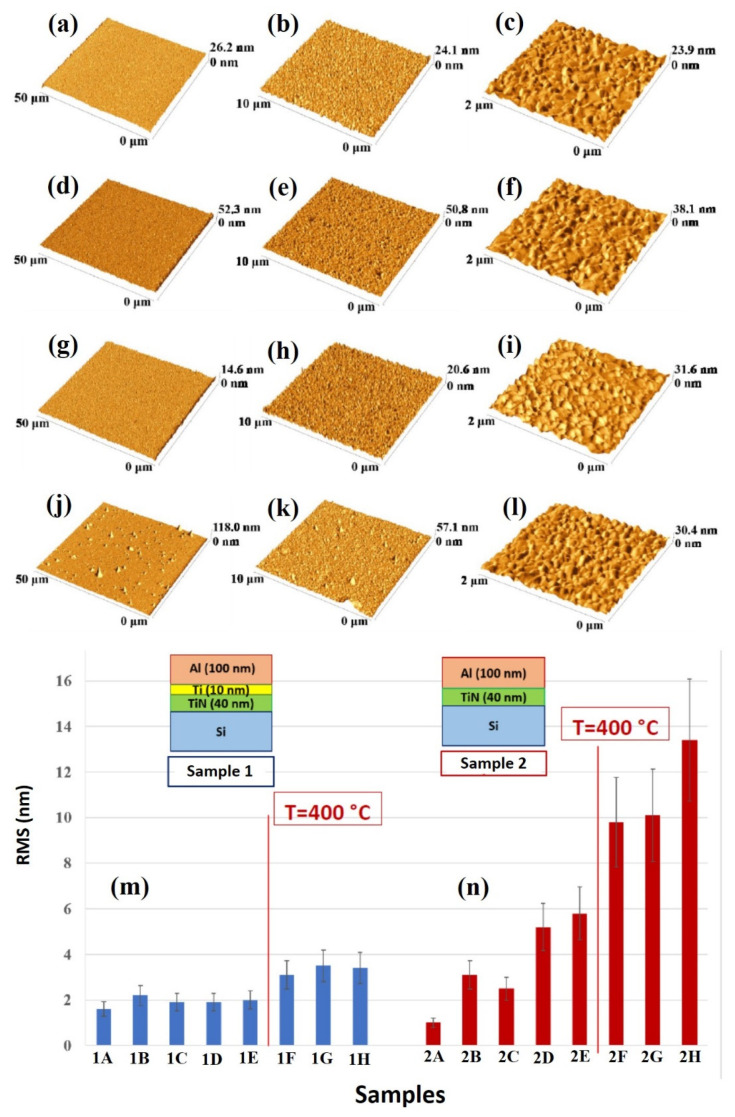
Representative AFM images (three-dimensional reconstructions) of the Al surface for the sample: (**a**–**c**) 1A (Si/TiN/Ti/Al) with decreasing scan size from (**a**) to (**c**); (**d**–**f**) 1H (Si/TiN/Ti/Al + 500 °C 15 min) with decreasing scan size from (**d**) to (**f**); (**g**–**i**) 2A (Si/TiN/Al) with decreasing scan size from (**g**) to (**i**); (**j**–**l**) 2H (Si/TiN/Al + 500 °C 15 min) with decreasing scan size from (**j**) to (**l**). (**m**) reports the Al layer surface roughness, as quantified by RMS through the AFM measurements and calculated by the 50 μm scan size images, for samples of class 1 (Si/TiN/Ti/Al) in the various thermal processing conditions. (**n**) reports the Al layer surface roughness, as quantified by RMS through the AFM measurements and calculated by the 50 μm scan size images, for samples of class 2 (Si/TiN/Al) in the various thermal processing conditions.

Figure 8 reports representative AFM images (three-dimensional reconstructions) of the Al surface for the sample: (a)–(c) 1A (Si/TiN/Ti/Al) with decreasing scan size from (a) to (c); (d)–(f) 1H (Si/TiN/Ti/Al + 500 °C 15 min) with decreasing scan size from (d) to (f); (g)–(i) 2A (Si/TiN/Al) with decreasing scan size from (g) to (i); (j)–(l) 2H (Si/TiN/Al + 500 °C 15 min) with decreasing scan size from (j) to (l). (m) reports the Al layer surface roughness, as quantified by RMS through the AFM measurements and calculated by using the 50 μm scan size images, for samples of class 1 (Si/TiN/Ti/Al) in the various thermal processing conditions. (n) reports the Al layer surface roughness, as quantified by RMS through the AFM measurements and calculated by using the 50 μm scan size images, for samples of class 2 (Si/TiN/Al) in the various thermal processing conditions.

**Figure 9:**
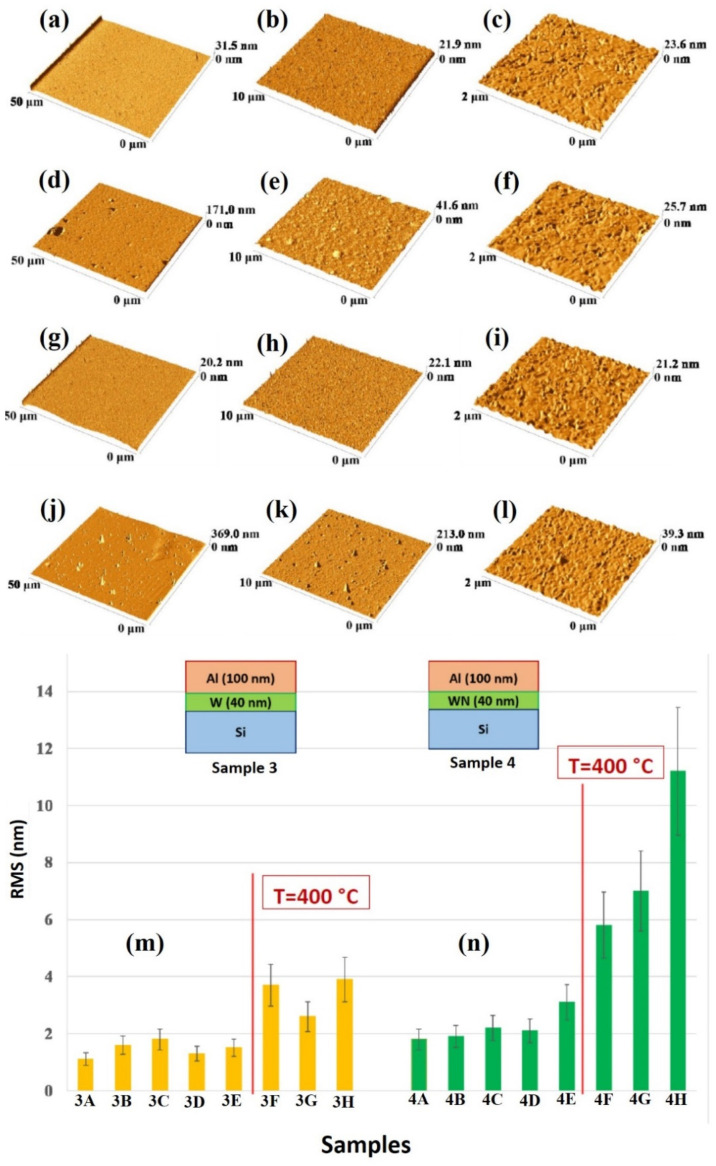
Representative AFM images (three-dimensional reconstructions) of the Al surface for the sample: (**a**–**c**) 3A (Si/W/Al) with decreasing scan size from (**a**) to (**c**); (**d**–**f**) 3H (Si/W/Al + 500 °C 15 min) with decreasing scan size from (**d**) to (**f**); (**g**–**i**) 4A (Si/WN/Al) with decreasing scan size from (**g**) to (**i**); (**j**–**l**) 4H (Si/WN/Al + 500 °C 15 min) with decreasing scan size from (**j**) to (**l**). (**m**) reports the Al layer surface roughness, as quantified by RMS through the AFM measurements and calculated by the 50 μm scan size images, for samples of class 3 (Si/W/Al) in the various thermal processing conditions. (**n**) reports the Al layer surface roughness, as quantified by RMS through the AFM measurements and calculated by the 50 μm scan size images, for samples of class 4 (Si/WN/Al) in the various thermal processing conditions.

Figure 9 shows representative AFM images (three-dimensional reconstructions) of the Al surface for the sample: (a)–(c) 3A (Si/W/Al) with decreasing scan size from (a) to (c); (d)–(f) 3H (Si/W/Al + 500 °C 15 min) with decreasing scan size from (d) to (f); (g)–(i) 4A (Si/WN/Al) with decreasing scan size from (g) to (i); (j)–(l) 4H (Si/WN/Al + 500 °C 15 min) with decreasing scan size from (j) to (l). (m) reports the Al layer surface roughness, as quantified by RMS through the AFM measurements and calculated by using the 50 μm scan size images, for samples of class 3 (Si/W/Al) in the various thermal processing conditions. (n) reports the Al layer surface roughness, as quantified by RMS through the AFM measurements and calculated by using the 50 μm scan size images, for samples of class 4 (Si/WN/Al) in the various thermal processing conditions. 

For each sample, the RMS parameter was calculated by using the 50 μm scan size images so as to draw information on the large-scale surface topography. On the other hand, the 2 μm scan size images allow us to recognize the granular morphology of the Al layer in agreement with the high-magnification SEM images. In particular, considering the plots in Figure 8m,n and Figure 9m,n, roughly, the critical annealing temperature can be seen to be 400 °C, from which the RMS value abruptly increases for each sample typology. However, this increase is higher in sample 2 than in sample 1. In addition, a slight increase of RMS in sample 2 starts at the annealing temperature of 350 °C. For both samples 3 and 4 the RMS increases starting from 400 °C.

The surface defects observed by SEM micrographs in Figure 4, Figure 6 and Figure 7 can be identified as standard pinholes and crater defects arising, during the annealing step, from processes such as grain growth, dislocation motion and possible Al-Si alloy formation [22,23]. For example, texture is one of the basic microstructural properties of Al poly-crystalline thin films and it is also related to the growth of hillocks and pinholes as activated by heat treatments and residual stress in thin films. This correlation was, for example, clearly observed in textured Al films grown on Ti-W substrates [22]. Hillocks and pinholes originate from the deposition process (growth hillocks and pinholes) and from the annealing process (annealing hillocks and pinholes). During the deposition process, heating from the heat flux originating from the sputtering target and/or from the nucleation process results in built-in intrinsic stress in the deposited layer which develops as the film grows. In turn, this can result in hillock growth or void formation, depending on process parameters such as sputter power or working gas pressure and temperature of post-deposition annealing processes. We observe, in our particular case, a strong effect of post-deposition annealing processes on the formation of pinholes in the Al layer, as evidenced from Figure 4, Figure 6 and Figure 7. Pinholes are one of the most common growth and annealing defects in physical-vapor-deposited thin films; they are discontinuities in the coating microstructure in the form of thin holes having a micron size diameter and extending from the substrate to the top surface of the coating. There are a number of mechanical and thermodynamic causes for formation of pinholes. A majority of pinholes are generated at the substrate imperfections, such as cavities (pits) or shallow depressions formed on the substrate surface. In our case, the influence of annealing temperature on the pinholes’ formation and growth is evident from the SEM images. However, possibly, by increasing the film thickness the stress would decrease, while the degree of crystallinity would increase, thus decreasing the surface density of the surface defects.

Hence, we can conclude, overall, that annealing processes T > 400 °C cause drastic degradation of the Al surface morphology in all the samples, which could be incompatible with successive growth processes on the Al surface towards the final design of the device. The surface morphology change of the Al surface, hence, consists of a surface roughening also compatible with the possible Al diffusion towards the Si substrate. However, to complete the characterizations, compositional analyses were performed by RBS measurements so as to draw information on the possible penetration of Al in the Si substrate through the diffusion barriers.

### 3.2. Elemental Analysis

RBS measurements were carried out in order to evaluate the efficiency of the various diffusion barriers in the samples and the possible interdiffusion between Al and Si.

**Figure 10:**
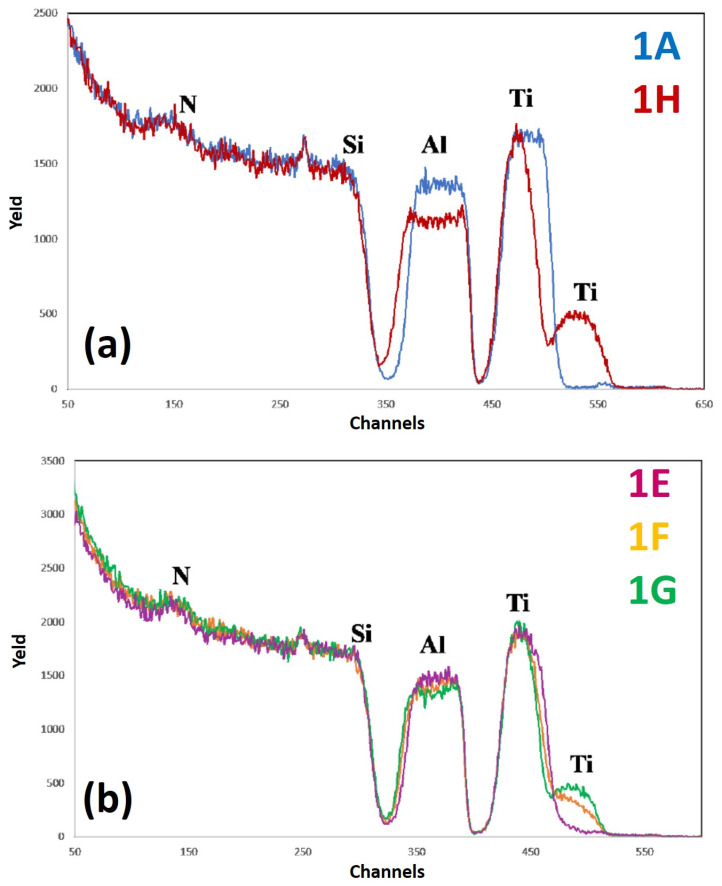
RBS spectra of the samples: (**a**) 1A (Si/TiN/Ti/Al)—blue curve, and 1H (Si/TiN/Ti/Al + 500 °C 15 min)—red curve; (**b**) 1E (Si/TiN/Ti/Al + 350 °C 30 min)—violet curve, 1F (Si/TiN/Ti/Al + 400 °C 15 min)—yellow curve, 1G (Si/TiN/Ti/Al + 450 °C 15 min)—green curve. The curves are reported in different plots to easily identify characteristic features and differences.

Figure 10 reports RBS spectra of the samples: (a) 1A (Si/TiN/Ti/Al)—blue curve, and 1H (Si/TiN/Ti/Al + 500 °C 15 min)—red curve; (b) 1E (Si/TiN/Ti/Al + 350 °C 30 min)—violet curve, 1F (Si/TiN/Ti/Al + 400 °C 15 min)—yellow curve, 1G (Si/TiN/Ti/Al + 450 °C 15 min)—green curve. The curves are reported in different plots to easily identify characteristic features and differences.

For each spectrum, the correspondence between characteristic peaks and the related element from which that peak arises is also indicated.

In particular, keeping in mind the structure of the sample (Figure 1a), the signal between channel 550 and channel 450 is due to the He ions backscattered by Ti atoms, the signal between channel 450 and channel 350 to the He ions backscattered by Al atoms, and the signal present at channels lower than channel 350 to the He ions backscattered by Si atoms. On the other hand, the peak at channel 250 is due to He ions backscattered by O atoms, while the peak at channel 150 can be associated to the He ions backscattered by N atoms. Considering, in particular, Figure 10a, comparing the RBS spectra of the as-deposited sample 1 and of the same sample annealed at the highest temperature (500 °C), it is clear that Si atoms have not diffused through the sample: on the contrary, in fact, a displaced Si signal should have been observed downward or with different slope in the region around channel 350. On the other hand, it is clear that a large amount of Ti atoms goes up in the Al layer and, conversely, some amount of Al atoms goes down below in the TiN layer.

This is evident from the Ti peak at channel 500 which is absent in the as-deposited sample while being present in the annealed sample and from the thickening and lowering of the Al signal. A mixed layer of Al and Ti is, thus, formed upon annealing (which should be a stable TiAl_3_ layer [5]) so that part of the Ti atoms reach the sample surface. It should be noted, however, that, as can be seen from the spectrum of sample 1A, there is some Ti contamination on the surface (small blue peak at channel 550), possibly due to an involuntary chamber contamination by Ti so that a very small amount of Ti is also deposited during the Al layer deposition. From Figure 10a, we can also observe a significant lowering of the Al signal, possibly due to the fact that part of Al atoms have migrated downward, similarly to the upward migration of Ti atoms. Moreover, considering Figure 10b, the lowering of the Al signal increases by increasing the annealing temperature, similar to how the thickening of the Ti signal increases by increasing the annealing temperature. At each annealing temperature, the shrinkage of the Ti signal is compensated for by the increase of the intensity of the Ti peak at the sample surface.

As a final conclusion, it can be suggested that the Ti/TiN layer does not act as a stable barrier for the Al and Si interdiffusion: from a chemical point of view, the sample undergoes significant changes upon annealing and, in particular, starting from the annealing temperature of 400 °C, the amount of the Ti atoms diffused to the sample surface is considerable. However, no interdiffusion between Al and Si is observed, meaning that the Ti/TiN layer fulfills its role as a “sacrificial barrier”, although without maintaining unaltered mechanical properties. In fact, by the AFM analysis, we also observed that the surface RMS of the sample significantly increases starting from the critical annealing temperature of 400 °C, which coincides with the temperature from which a considerable amount of Ti atoms migrate to the sample surface.

**Figure 11:**
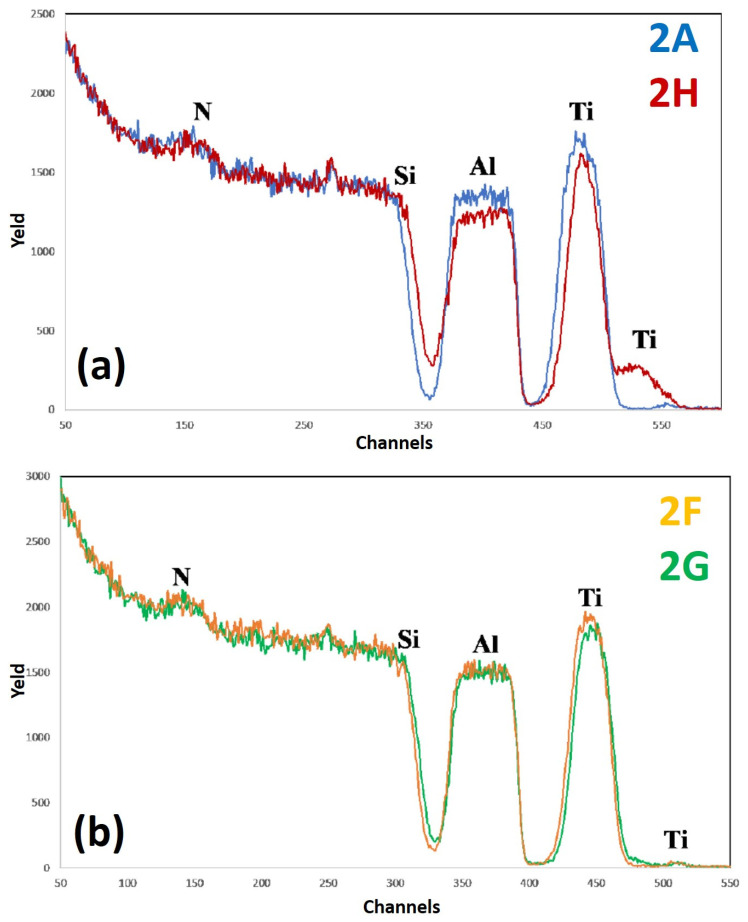
RBS spectra of the samples: (**a**) 2A (Si/TiN/Al)—blue curve, and 2H (Si/TiN/Al + 500 °C 15 min)—red curve; (**b**) 2F (Si/TiN/Al + 400 °C 15 min)—yellow curve, 2G (Si/TiN/Al + 450 °C 15 min)—green curve. The curves are reported in different plots to easily identify characteristic features and differences.

Figure 11 reports RBS spectra of the samples: (a) 2A (Si/TiN/Al)—blue curve, and 2H (Si/TiN/Al + 500 °C 15 min)—red curve; (b) 2F (Si/TiN/Al + 400 °C 15 min)—yellow curve, 2G (Si/TiN/Al + 450 °C 15 min)—green curve. The curves are reported in different plots to easily identify characteristic features and differences. For each spectrum, the correspondence between characteristic peaks and the related element from which that peak arises is also indicated. In this case, for sample 2, with respect to sample 1, the additional layer of Ti is missing between Al and TiN.

In particular, the RBS spectra of sample 2A (as-deposited) and 2H (as-deposited annealed at 500 °C for 15 min) are shown in Figure 11a, while the RBS spectra corresponding to sample 2F (as-deposited annealed at 400 °C for 15 min) and 2G (as-deposited annealed at 450 °C for 15 min) are reported in Figure 11b. Comparing the spectra in Figure 11a, we can observe that the Ti peak tightens and lowers, indicating the migration of Ti atoms to the surface of the sample. This phenomenon is confirmed by the significant increase of the intensity of the superficial Ti peak. Moreover, it can be recognized that the signal of Al is significantly lowered, while that of Si moves towards higher channels. These two facts indicate the thinning of the Al layer. In fact, the slope of the Si peak does not change upon annealing and, therefore, it cannot be stated that diffusion of Ti atoms into the Si substrate occurred. For annealing temperature below 500 °C (see Figure 11b) the spectra are similar between them, apart from a slight narrowing and shift to the right of the Ti peak and a consequent slight increase of the surface Ti signal. Finally, hence, the TiN diffusion barrier alone does not seem to be an excellent barrier, and is worse than the TiN/Ti combined layers: although there is a significant diffusion of Ti on the surface only at the extreme temperature of 500 °C, unlike sample 1 in which this occurs starting from 400 °C, in this case a slight advance of the Si signal is noted in sample 2H, which is not observed in sample 1. This is due to the fact that the TiN layer, after 500 °C, has become excessively thin. Furthermore, the RMS value for the Al surface is significantly higher than those of sample 1. We recall that SEM images of the surface of sample 2H clearly evidenced some characteristic features compatible with the formation of holes distributed over the sample surface and determining the significant increase of the surface roughness upon the 500 °C annealing. We can state, overall, that the barrier of sample 1, consisting of Ti/TiN layers, is better than that of sample 2, consisting only of TiN layer, at least from a mechanical point of view.

**Figure 12:**
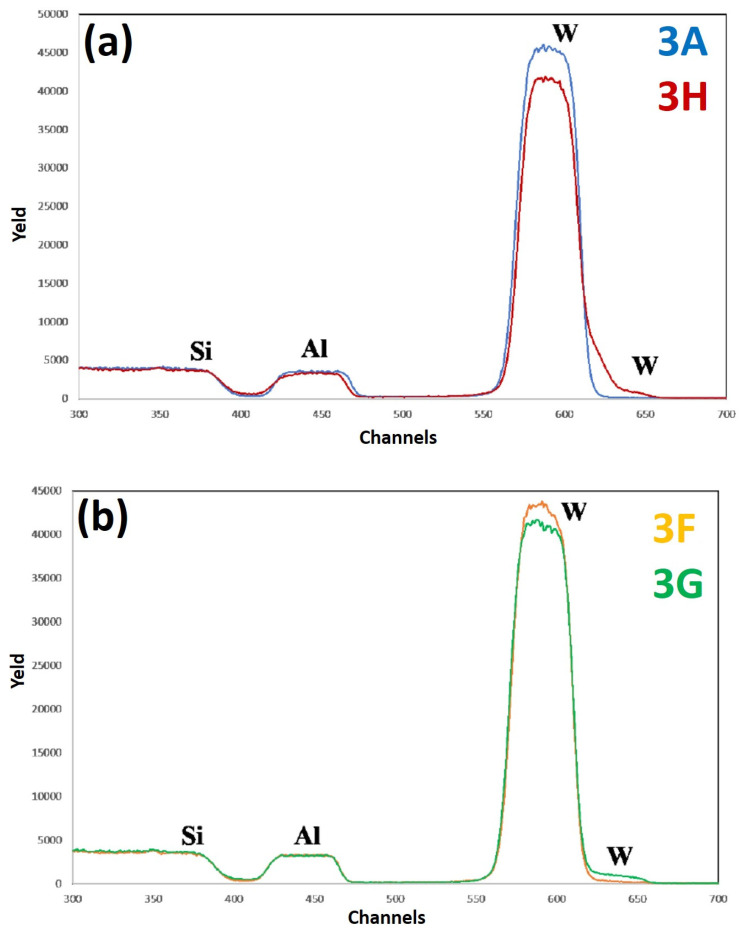
RBS spectra of the samples: (**a**) 3A (Si/W/Al)—blue curve, and 3H (Si/W/Al + 500 °C 15 min)—red curve; (**b**) 3F (Si/W/Al + 400 °C 15 min)—yellow curve, 3G (Si/W/Al + 450 °C 15 min)—green curve. The curves are reported in different plots to easiliy identify characteristic features and differences.

Figure 12 reports RBS spectra of the samples: (a) 3A (Si/W/Al)—blue curve, and 3H (Si/W/Al + 500 °C 15 min)—red curve; (b) 3F (Si/W/Al + 400 °C 15 min— yellow curve, 3G (Si/W/Al + 450 °C 15 min)—green curve. The curves are reported in different plots to easily identify characteristic features and differences. For each spectrum, the correspondence between characteristic peaks and the related element from which that peak arises is also indicated. Figure 12a compares the RBS spectra of the as-deposited sample (3A) and of the as-deposited sample after 500 °C annealing for 15 min (3H). Figure 12b compares the RBS spectra for the samples annealed at 400 °C for 15 min (3F) and at 450 °C for 15 min (3G). Upon the 500 °C annealing, a significant lowering of the W peak followed by a corresponding increase in the W signal at the surface can be observed. Moreover, at lower annealing temperature this phenomenon occurs (see the spectrum of sample 3G), albeit to a lesser extent. Even though there is a lowering of the W peak in sample 3H, however, the superficial part of the Al layer is not affected.

**Figure 13:**
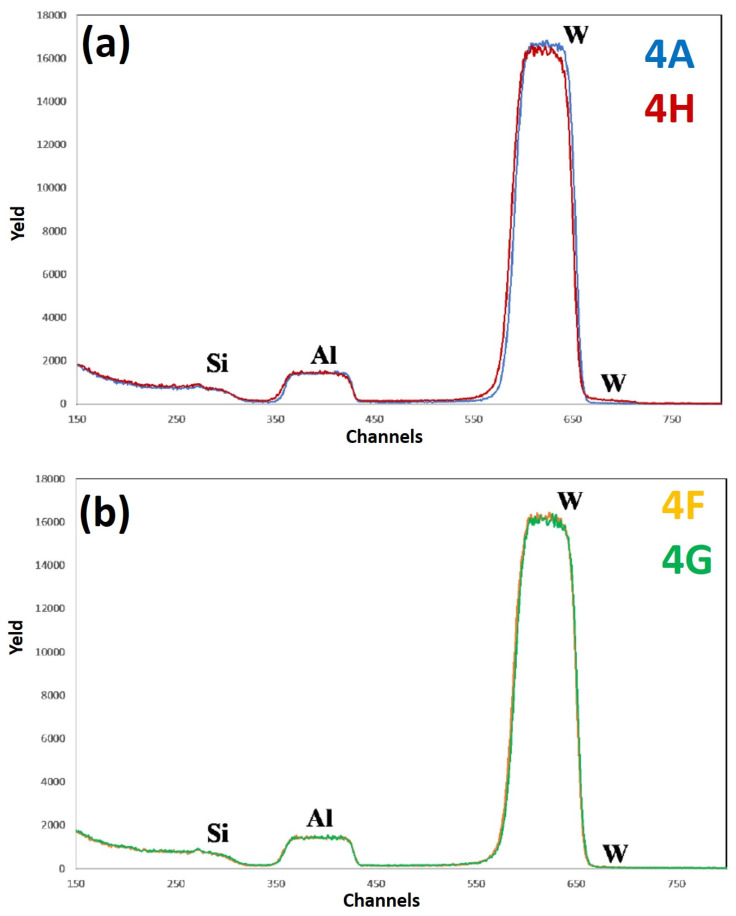
RBS spectra of the samples: (**a**) 4A (Si/WN/Al)—blue curve, and 4H (Si/WN/Al + 500 °C 15 min)—red curve; (**b**) 4F (Si/WN/Al + 400 °C 15 min)—yellow curve, 4G (Si/WN/Al + 450 °C 15 min)—green curve. The curves are reported in different plots to easily identify characteristic features and differences.

Figure 13 reports RBS spectra of the samples: (a) 4A (Si/WN/Al)—blue curve, and 4H (Si/WN/Al + 500 °C 15 min)—red curve; (b) 4F (Si/WN/Al + 400 °C 15 min)—yellow curve, 4G (Si/WN/Al + 450 °C 15 min)—green curve. The curves are reported in different plots to easily identify characteristic features and differences. For each spectrum, the correspondence between characteristic peaks and the related element from which that peak arises is also indicated. Comparing the RBS spectra from sample 4A (as-deposited) to sample 4G (as-deposited annealed at 450 °C for 15 min), the WN layer appears to be the best diffusional barrier for Al and Si, in comparison to the other analyzed systems. The spectra of samples 4F and 4G are perfectly superimposed, not showing particular compositional variations up to a temperature of 450 °C. However, comparing the RBS spectra of samples 4A and 4H, a significant difference can be noted. Upon annealing at 500 °C, it is evident that the W is rising along the layer of Al, but not so much as to raise to the surface; in fact, the signal of W on the surface is almost zero, confirming the fact that the W remains located under the Al layer (i.e., no significant migration of W atoms towards the Al surface). The Al-WN interface is, surely, no longer sharp and a certain amount of W enters in the Al layer (as evidenced by the upward shift of the W signal); however, W atoms do not reach the Al surface. In the spectrum of sample 4H, we can observe that the Al signal moves slightly downwards and that the W starts to drop through the Al signal. We can conclude that only a minimal part of the Al layer is affected by the ascent of the W, which, however, does not reach the Al surface. Comparing the RBS results for samples 3 and 4, we can state that the WN layer acts as a better diffusional barrier with respect to the W barrier alone. This is confirmed by some literature data [18], according to which the addition of N during the deposition of W thin films, by saturating the W grain boundaries, determines the inhibition of Si and Al atoms’ diffusion through the grain boundaries. In general, the superior performance of the WN layer as diffusion barrier between Al and Si can be attributed to the following crossed factors: (a) a superior barrier density so that a more dense structure, formed by smaller grains, inhibits Al and Si diffusion [8]; (b) the incorporation of N which, saturating the grain boundaries, inhibits Al and Si grain boundaries diffusion [8]—in general, metal nitrides are more chemically stable than their originated metal [18]; (c) the absence of Ti—a main failure mechanism for barrier layers containing Ti is often ascribed to Ti diffusion into the Al layer from an annealing temperature of 400 °C [8]; (d) superior mechanical and thermal properties resulting from an intrinsic stress as well as from thermal mismatch between adjacent materials; Al penetrates easily through microcracks in the barrier, which enhances the Al-Si reaction and causes a barrier failure [4]. These effects are inhibited by the superior mechanical and thermal properties of the WN layers. However, despite the higher efficiency shown by the WN layer in inhibiting the Al-Si interdiffusion, we have to observe that the surface roughness (RMS) of sample 4 is greater than that of sample 3, by increasing the annealing temperature. In fact, SEM images of samples 4G, 4F, and 4H show the formation of surface inhomogeneities upon annealing, compatible with the formation of holes in the Al layer, which grow in size and number as the annealing temperature increases.

To conclude, it is worthy of mention that the present work compares four different systems and reports detailed comparisons regarding morphological and compositional characterizations changing processing parameters (annealing temperature), although in fixed designed structural configurations (diffusion barrier layer thickness). Therefore, we consider this work as a starting study which forms the basis for further extended investigations. In this sense, important perspectives for future investigations are: 

(a) The extension of diffusion barrier studies by changing the thickness of the barrier layers: maintaining the barrier layers’ (TiN, W, WN) thickness in the nanometer range (so as to hold the potential applications in ultra-scaled Si-based devices), it will be important to repeat the investigations on the performances of these layers as diffusion barriers between Al and Si by changing the layers’ thickness (20 nm and 60 nm in addition to the existing study 40 nm-thick films). These studies will allow us to define the best thickness condition, maximizing the diffusion barrier performance. On the one hand, we can expect that by increasing the barrier thickness, the formation of defects in the barrier layers should be progressively reduced and improving, and consequently, the barrier performance will also improve. On the other hand, it is also important to test the possibility of reducing the barrier layers’ thickness till a limit at which the barriers’ properties are not dramatically worsened with respect to the 40 nm-thick conditions. Crossing the corresponding results, it will be possible to establish the condition of a technologically suitable performance of the layers as diffusion barriers between Al and Si maintaining, possibly, very thin barrier layers.

(b) The extension of the structural characterizations of the systems by X-ray diffraction and/or electron diffraction analysis. The results of these studies should provide information on the crystallinity of the diffusion barrier layers versus layer thickness and annealing temperature. These results will help in establishing an additional correlation between the structure of the diffusion barrier layers and their performance as blocking layers for the Al-Si interdiffusion. In fact, these analyses should provide a means to identify the main diffusional path for Al and Si (i.e., grain boundary diffusion, volume diffusion, etc.).

(c) The extension of the compositional analysis of the systems by secondary ions mass spectrometry and/or scanning transmission electron microscopy. These characterizations, performed versus the barrier layer thickness and annealing time, should provide information, with atomic resolution, on the spatial distribution of Al and Si within the barrier layers. The results, combined with the diffraction analysis, should help in the identification of the main diffusion mechanism and in the identification of additional strategies to improve the performances of the used layers as diffusion barriers between Al and Si.

(d) The extension of the analysis of the diffusion barrier properties of the Ti/TiN, TiN, W, WN layers deposited by different techniques, as laser-based deposition methods, so as to provide information on the barriers’ performances as resulting from the fabrication approach in addition to the standard sputtering deposition and evaporation deposition techniques. In fact, the characteristics of the specific deposition technique can affect the structural characteristics of the deposited layer, thus affecting the diffusional mechanisms between Al and Si.

## 4. Conclusions

In the present work, an investigation of the properties of nanoscale-thick Ti/TiN (sample 1), TiN (sample 2), W (sample 3), WN (sample 4) layers as diffusion barriers between Si and Al were investigated. Each sample was subjected to heat treatments at varying time and temperature to activate interdiffusion between Si and Al. Each sample was morphologically characterized by SEM and AFM analysis.

Regarding sample 1, no particular morphological changes of the Al surface film were found; as the temperature varied, a significant increase of the RMS starting from a critical temperature of 400 °C was observed. In addition, a significant migration of Ti atoms towards the surface, through the Al layer, starting from the temperature of 400 °C was observed. However, interdiffusion between Al and Si was not observed.

Regarding sample 2, the formation of surface holes was observed alongside a roughening of the surface starting from a temperature of 400 °C. Moreover, a significant migration of Ti towards the surface at a temperature of 500 °C was measured. However, interdiffusion between Al and Si was not observed.

Regarding sample 3, the formation of surface defects, starting from the annealing temperature of 450 °C, was evidenced alongside a significant RMS increase starting from a temperature of 400 °C. Furthermore, a significant migration of W atoms towards the surface, through the Al layer, starting from the temperature of 450 °C, was observed. However, interdiffusion between Al and Si was not observed.

Regarding sample 4, the WN layer seems to have the best diffusion barrier characteristics, as the migration of W atoms through the Al layer is minimal till 450 °C. Despite this fact, however, a significant increase of the surface roughness was observed by increasing the annealing temperature alongside the formation of surface inhomogeneities upon annealing. This could be due to the thermal evaporation technique used to deposit the WN layer: several studies [18,19,20] have shown that the deposition of WN by thermal evaporation does not guarantee a uniform coating of the substrate and that it generates microfractures and micro-holes in the film which are amplified by subjecting the sample to subsequent heat treatments. In conclusion, among the Ti/TiN, TiN, W, WN layers the best in terms of diffusion barrier, considering merely the inhibition of diffusion and the consequent interdiffusion between Al and Si, is the WN one. However, from the mechanical properties point of view, the sample containing the WN layer also presents a significant increase of the surface roughness upon annealing which could be, in perspective, solved by changing the layer thickness or the deposition technique. All these results are also connected to the materials science findings for semiconductor device fabrication: as already stated, the continuous shrinking of the geometrical dimension of ultra-large-scale-integrated circuits causes severe problems in conventional Al metallization. In general, the step coverage of physical vapor deposited films (such as sputter deposition or evaporation) is inferior to that of chemical vapor deposited films [18]. Moreover, residual stress generated during the sputtering process often causes microcracks or microvoids in the films. This combined with poor step coverage and poor control of composition can cause fatal barrier failure in physical vapor deposited diffusion barriers. On the other hand, sputtering and evaporation-based deposition techniques are better-suited for large-scale industry production. Therefore, the findings of the present work contribute to the optimization of sputter-deposited and evaporated chemically stable and efficient materials layers to contrast the Al-Si interdiffusion.

## Figures and Tables

**Table 1:** Summary of the combinations of annealing temperature and time used to process the samples and description of the corresponding labels which were associated to the samples after the annealing processes.

	T (°C)	325	350	400	450	500
t (min)						
15		1B, 2B, 3B, 4B	1D, 2D, 3D, 4D	1F, 2F, 3F, 4F	1G, 2G, 3G, 4G	1H, 2H, 3H, 4H
30		1C, 2C, 3C, 4C	1E, 2E, 3E, 4E			
